# Two Argan Oil Phytosterols, Schottenol and Spinasterol, Attenuate Oxidative Stress and Restore LPS-Dysregulated Peroxisomal Functions in *Acox1^−/−^* and Wild-Type BV-2 Microglial Cells

**DOI:** 10.3390/antiox12010168

**Published:** 2023-01-11

**Authors:** Soukaina Essadek, Catherine Gondcaille, Stéphane Savary, Mohammad Samadi, Joseph Vamecq, Gérard Lizard, Riad El Kebbaj, Norbert Latruffe, Alexandre Benani, Boubker Nasser, Mustapha Cherkaoui-Malki, Pierre Andreoletti

**Affiliations:** 1 Laboratory of Biochimistry, Neuroscience, Natural Resources and Environment, Faculty of Science and Technology, University Hassan I, Settat 26000, Morocco; ess.soukaina@hotmail.fr (S.E.); boubker.nasser@uhp.ac.ma (B.N.); 2 Bio-PeroxIL Laboratory, EA7270, University Bourgogne Franche-Comté/Inserm, 6 Boulevard Gabriel, 21000 Dijon, France; catherine.gondcaille@u-bourgogne.fr (C.G.); stsavary@u-bourgogne.fr (S.S.); gerard.lizard@u-bourgogne.fr (G.L.); norbert.latruffe@u-bourgogne.fr (N.L.); 3 Laboratory of Chemistry and Physics Multi-Scale Approach to Complex Environments, Department of Chemistry, University Lorraine, 57070 Metz, France; mohammad.samadi@univ-lorraine.fr; 4 Inserm and HMNO, CBP, CHRU Lille, and RADEME EA 7364, Faculté de Médecine, Université de Lille 2, 59045 Lille, France; joseph.vamecq@inserm.fr; 5 Laboratory of Health Sciences and Technologies, Higher Institute of Health Sciences, Hassan 1st University, Settat 26000, Morocco; elkebbajriad@gmail.com; 6 CSGA—Centre des Sciences du Goût et de l’Alimentation, CNRS—Centre National de la Recherche Scientifique, INRAE—Institut National de Recherche pour L’agriculture, L’alimentation et L’environnement, Institut Agro Dijon, University Bourgogne Franche-Comté, 21000 Dijon, France; alexandre.benani@u-bourgogne.fr

**Keywords:** Acyl-CoA oxidase 1, argan oil, BV-2, catalase, inflammation, LPS, microglia, peroxisome, schottenol, Spinasterol

## Abstract

Oxidative stress and inflammation are the key players in neuroinflammation, in which microglia dysfunction plays a central role. Previous studies suggest that argan oil attenuates oxidative stress, inflammation, and peroxisome dysfunction in mouse brains. In this study, we explored the effects of two major argan oil (AO) phytosterols, Schottenol (Schot) and Spinasterol (Spina), on oxidative stress, inflammation, and peroxisomal dysfunction in two murine microglial BV-2 cell lines, *wild-ype* (*Wt*) and *Acyl-CoA oxidase 1* (*Acox1*)-deficient cells challenged with LPS treatment. Herein, we used an MTT test to reveal no cytotoxicity for both phytosterols with concentrations up to 5 µM. In the LPS-activated microglial cells, cotreatment with each of these phytosterols caused a significant decrease in intracellular ROS production and the NO level released in the culture medium. Additionally, Schot and Spina were able to attenuate the LPS-dependent strong induction of *Il-1β* and *Tnf-α* mRNA levels, as well as the *iNos* gene and protein expression in both *Wt* and *Acox1^−/−^* microglial cells. On the other hand, LPS treatment impacted both the peroxisomal antioxidant capacity and the fatty acid oxidation pathway. However, both Schot and Spina treatments enhanced ACOX1 activity in the *Wt* BV-2 cells and normalized the catalase activity in both *Wt* and *Acox1^−/−^* microglial cells. These data suggest that Schot and Spina can protect cells from oxidative stress and inflammation and their harmful consequences for peroxisomal functions and the homeostasis of microglial cells. Collectively, our work provides a compelling argument for the protective mechanisms of two major argan oil phytosterols against LPS-induced brain neuroinflammation.

## 1. Introduction

Microglial cells play a sentinel role in the regulation of brain development and homeostasis [1]. They are involved in oxidative stress; neuroinflammation; and the physiopathogenesis of several neurodegenerative diseases, including Alzheimer’s disease, multiple sclerosis, and peroxisomal leukodystrophies [2,3]. Activated microglia may disturb healthy neurons, leading to neurodegeneration [4], by producing proinflammatory molecules, including tumor necrosis factor-α (TNF-α), interleukin (IL)-1, IL-6, reactive oxygen species (ROS), and nitric oxide (NO) [5,6,7]. The activation of microglia can also be triggered by lipopolysaccharides (LPS) in the brain of animal models for sepsis [8,9]. Furthermore, LPS-activated microglia can trigger the death of growing oligodendrocytes [10]. The release of ROS by LPS-activated microglia is a key step in the generation of neurotoxicity [11], which can be principally abrogated by neutralizing extracellular hydrogen peroxide and superoxide by catalase and superoxide dismutase treatment, respectively [12,13]. This underlines the key antioxidant function of peroxisomal antioxidant enzymes.

Peroxisomal leukodystrophies include peroxisome biogenesis disorders caused by a mutation of one of the peroxin-encoding genes, leading to the Zellweger syndrome spectrum [14,15]. On the other hand, some inborn errors only concern the transport or the metabolism of very-long-chain fatty acids (VLCFA). For instance, X-linked adrenoleukodystrophy is associated with mutations in the ATP-binding cassette transporter D1 (*ABCD1*) gene and ACOX1 deficiency with mutations in the acyl-CoA oxidase 1 (*ACOX1*) gene, which controls the rate-limiting step of peroxisomal β-oxidation [16]. In these leukodystrophies, increased oxidative stress, associated with peroxisomal metabolism dysfunction, is now considered the first stage in the development of a progressive demyelination and neurodegeneration [17]

With the aim of deciphering the role of microglia in the physiopathogenesis of the peroxisomal leukodystrophies, we used CRISPR/Cas9 gene editing to generate microglial cell models deficient in the peroxisomal VLCFA β-oxidation pathway [18,19]. Among these cell models, the microglial *BV-2-Acox1^−/−^* cell line exhibited a substantial change in the expression of several key genes involved in microglial functions related to antioxidant activity, inflammation, and phagocytosis [19]. Furthermore, we recently reported that argan oil (AO) pretreatment can abrogate the early oxidative stress caused by LPS and preserve peroxisomal functions, including antioxidant and β-oxidation activities, in both brain and liver mouse tissues [20]. AO has been shown to attenuate the oxidative stress; organelle dysfunction (mitochondria, lysosomes, peroxisomes); and cell death in the oligodendrocytes caused by pro-oxidant compounds such as 7-ketocholesterol, which is often increased during aging and in patients with age-related diseases [21]. In addition, the AO phytosterols, Schottenol (Schot) and Spinasterol (Spina), are able to modulate the mitochondrial membrane potential of microglial BV-2 cells [22].

Phytosterol-enriched foods have been marketed for decades and have gained much attention in the last few years [23]. They belong to the triterpene family with a cholesterol-like structure and are present at high concentrations in edible vegetable oils [24]. The total phytosterol content varies between 83 and 160 mg/100 g of argan oil, which contains the following major sterols (in mg/100g oil): α-cholestanol (12.63%), compestanol (3.73%), campesterol (0.2%), Δ-7-Avenasterol (4.2%), β-Sitosterol (3.56%), Spinasterol (35.3%), and Schottenol (43,8%) [22,25]. Phytosterols are widely used as food supplements, and it has been claimed that they are highly beneficial to humans in terms of health and disease [26]. The pharmacological properties of plant phytosterols for human health have been investigated in several studies [27]. Phytosterols interfere with the intestinal absorption of cholesterol, leading to a reduction in blood cholesterol levels [24,28,29], which underlies their benefit of lowering the risk of cardiovascular disease [30]. Phytosterols have also displayed significant anti-inflammatory, antibacterial, antiulcerative, and antitumor properties [27,31,32]. These compounds reduced the expression of the pro-inflammatory mediators (i.e., cyclooxygenase-2 and nitric oxide synthase (iNOS)) in LPS-stimulated RAW264.7 macrophages [33].

Although the antioxidant and anti-inflammatory effects of AO have been well-investigated, to date, few studies have explored the compounds responsible for its biological activities. Here, we attempted to investigate the antioxidant and anti-inflammatory effects, as well as the capacity to restore peroxisomal functions, of two major AO phytosterols derivatives, Schottenol (Schot) and Spinasterol (Spina). In this study, we investigated their effects on both wild-type (*Wt*) and ACOX1-deficient (*Acox1^−/−^*) BV-2 cell lines [19], with and without activation by LPS.

## 2. Materials and Methods

### 2.1. Cell Cultures and Treatments

The wild-type (*Wt*) [34] and knockout *Acox1* (*Acox1^−/−^*) [19] BV-2 microglial murine cell lines were grown and maintained in Dulbecco’s modified Eagle’s medium (DMEM, Lonza, Amboise, France) supplemented with 10% fetal bovine heat-inactivated serum (Dutscher, Bernolsheim, France) and 1% penicillin/streptomycin antibiotics (Dutscher, Bernolsheim, France). Cells were cultured at an appropriate seeding density at 37 °C in a humidified incubator under 5% CO_2_. Cells were trypsinized in 0.05% trypsin−0.02% EDTA solution.

Schot and Spina were synthesized by Mohammad Samadi (Department of Chemistry, University Lorraine, Metz, France) and previously characterized [35]. Five milligrams of Schot or Spina was diluted in 500 µL of absolute ethanol; afterwards, the solutions were sonicated for 10 min then stored at 4 °C until use, at which point they were diluted in the culture medium to achieve the final concentrations indicated in the figures. 7-ketocholesterol (7-KC), known for its cytotoxicity, was used as a positive control in the MTT assay. Stock solution was prepared by dissolving 5 mg of 7-KC in 250 µL of absolute ethanol and then diluted in the culture medium to achieve a final concentration of 20 µM.

### 2.2. MTT Test

An MTT test was used to evaluate the cytotoxicity of Spina and Schot at 24 h or 48 h of treatment on BV2-*Wt* and BV2-*Acox1^−/−^* cell proliferation and/or viability [36]. We seeded 4 × 10^4^ cells/well in 24-well plates and treated them for 24 h or 48 h with Spina at 1, 2.5, 5, or 20 µM; Schot at 1, 2.5, 5, or 20 µM; or 7-KC at 20 µM for 24 h or 48 h as a positive control [22]. At the end of treatment, cells were incubated for 3 h with an MTT solution at a final concentration of 0.05 mg/mL. After incubation, 1 ml of DMSO/well was added to dissolve the formazan crystals. Absorbance was measured at 595 nm.

### 2.3. Griess Test

Nitric oxide production in the culture medium was measured using the Griess reagent test [37]. After 24h incubation at 4 × 10^4^ cells/well with different treatments (Schot at 1 or 2.5 µM and Spina at 1 or 2.5 µM), activation was performed with LPS (1 µg/mL). The supernatant was removed and placed in a 96-well plate; an equal volume of Griess reagent (1% sulphanilamide and 0.1% naphthyl-ethylene-diamine in 5% H_3_PO_4_) was added after 30 min incubation in the dark at room temperature; and the absorbance was measured at 540 nm. For the calibration curve, NaNO_2_ dilutions from 0.39 to 100 µM were used.

### 2.4. Measurement of Intracellular ROS

Intracellular ROS production levels were measured using 2′,7′-dichlorodihydrofluorescein diacetate (H_2_DCFDA) and dihydroethidium (DHE) assays [38]. Cells were seeded at a density of 2 × 10^4^ cells/well in a 96-well plate in DMEM phenol-red-free medium for 7 h (the peak of ROS production after LPS stimulation, according to a personal communication with Stephane Savary) with different phytosterol treatments with or without LPS stimulation. Then, the treatment medium was discarded, and cells were washed with PBS and incubated with either 5 μM H_2_DCFDA or 10 μM DHE for 70 min at 37 °C in 5% CO_2_. Cell fluorescence emissions were measured using a fluorescence spectrophotometer (Tecan Infinite Pro M200, Tecan, Lyon, France). Hoechst staining was used to estimate cell density and calculate ROS production per cell using the formulas below.

For H_2_DCFDA: R_1_ = (mean H_2_DCFDA-background)/(mean Hoechst–background)

For DHE: R_2_ = (mean DHE-background)/(mean Hoechst–background)

The reported values are ratios calculated as R treated cells/R control untreated cells.

### 2.5. Cell Homogenate Preparation

We rinsed 8 × 10^6^ BV-2 cells in PBS and then lysed them in RIPA lysis buffer (Tris 1M, NaCl 150 mM, NP-40 at 1%, SDS 0.1%, and sodium deoxycholate 1%) containing a mixture of protease inhibitors. Cells were homogenized on ice using an ultrasound and then centrifuged for 30 min at 10,000× *g*. The supernatants were collected, and the protein content was measured using bovine serum albumin as a standard and a Bicinchoninic Acid Kit (SigmaAldrich, Saint-Quentin-Fallavier, France). Samples were stored at −80 °C until use for further experiments.

### 2.6. Catalase Activity Measurement

Catalase activity was evaluated following a photometric measurement of the H_2_O_2_ decomposition by catalase contained in the cell extract at 240 nm [39]. The reaction was carried out in a special 96-well UV microplate (Greiner 655801 type, Dutscher, Bernolsheim, France France). A reaction mixture of H_2_O_2_ solution (30%) and Tris-HCl buffer (1M, pH 7.4) was added to the cell extract, and the reaction was carried out for 2 min. One unit of the enzyme is defined as 1 µmol of H_2_O_2_ consumed per minute, and the specific activity is reported as units per milligram of protein according to the following formula:

$$\mathrm{Catalase}\;specific\;activity=\frac{\Delta Abs_{240\mathrm{nm}}\cdot min^{-1}}{43.2}\times 10^{6}\times \frac{200}{10}\times \frac{1}{\left[protein\right]mg\cdot l^{-1}}$$

### 2.7. Acyl-CoA Oxidase 1 Activity Measurement

The measurement of ACOX1 activity was performed according to the protocol described by Oaxaca-Castillo et al. [40] using a fluorometry-based assay. The reaction was carried out in a 96-well plate using 200 µL reaction mixture containing Tris-buffer (50 mM, pH 8.3); horseradish peroxidase (20 mg/mL); homovanillic acid (0.75 mM); and acyl-CoA substrate (palmitoyl-CoA at a final concentration of 50 mM). The reaction was initiated at 30 °C by the addition of 10 µL enzymatic solution. The kinetics of the appearance of fluorescence was measured at 420 nm every 30 s for 120 cycles (total kinetic of 60 min) using a fluorimeter (Fluorimeter/luminometer Infinite M200 pro, TECAN). The initial rate of the reaction was determined from the kinetic curves, and the calculation of the specific activity of ACOX1 is expressed in units of H_2_O_2_ produced per minute per milligram of protein (1 μM d’H_2_O_2_ corresponds to 115.75 RFU) according to the following formula:

(1) $$\mathrm{ACOX}1\;specific\;activity=\frac{\Delta RFU_{}\cdot min^{-1}}{RFU\;\left(correspond\;of\;1{\;\mu M\;d\prime H}_{2}O_{2}\;\right)\cdot min^{-1}}\times Fd\times \frac{200}{10}\times \frac{1}{\left[protein\right]mg\cdot l^{-1}}$$

### 2.8. Immunoblotting

Cell protein lysate was prepared as described above. Fifty micrograms of protein was diluted (*v*/*v*) in the loading buffer (125 mM Tris-HCl, pH 6.8, 4% SDS, 20% glycerol, 14% mercaptoethanol, and 0.003% Bromophenol blue), and the complete denaturation of the protein was achieved by incubation at 100 °C for 5 min. Samples were then separated on a 10% SDS-PAGE gel and transferred onto a PVDF membrane. The non-specific binding sites were blocked with 5% nonfat milk in TBST (10 mM Tris-HCl, 150 mM NaCl, 0.1% Tween 20, pH 8) for 1 h at room temperature. The membrane was incubated with the primary antibody diluted in 1% milk TBST overnight at 4 °C (anti-ABCD1,“serum 029” from Bio-PeroxIL laboratory [18], dilution 1/2000; anti-ABCD2, ab 102948, from Abcam, Paris, France, dilution 1/1000; anti-catalase, AF3398 from R&D Systems Noyal Châtillon sur Seiche, France, dilution 1/400; anti-β-actin, A2228 from Sigma-Aldrich, Saint-Quentin-Fallavier, France, dilution 1/10,000). Following three washes for 10 min in PBST, the membranes were immersed for 1 h at room temperature with an appropriate secondary antibody (dilution 1/5000 in 1% milk TBST) conjugated to horseradish peroxidase. Membranes were washed three times in TPBS for 10 min, and the Supersignal West Femto Maximum Sensitivity Substrate (ThermoFisher Scientific, Illkirch-Graffenstaden, France) was used to reveal the immunoreactivity through enhanced chemiluminescence and a Chemidoc XRS+ device (Bio-Rad, Marnes-la-Coquette, France). Image processing and quantification were performed using Image Lab software (Bio-Rad, Marnes-la-Coquette, France).

### 2.9. Quantitative Reverse-Transcription-PCR

We used RT-qPCR to determine the mRNA expression in the *Wt* and *Acox1^−/−^* BV-2 microglia cell lines after different treatments. Cells were collected by trypsinization (trypsin-EDTA, 2 mM solution) and then washed twice with PBS after centrifugation for 5 min at 300 g. Cell pellets were used for total RNA extraction and purification using the RNeasy Mini kit (Qiagen, Valencia, CA, USA) following the manufacturer’s instructions. The purity of nucleic acids was controlled by the ratio of absorbance at 260 nm to 280 nm, accepting a ratio between 1.8 and 2.2. cDNA was generated by reverse-transcription using an iScript cDNA Synthesis Kit (Bio-Rad) according to the manufacturer’s instructions. The quantitative PCR of cDNA was realized using FG Power SYBR Green (Thermo Fischer Scientific, Illkirch-Graffenstaden, France) and an iCycler iQ Real-Time Detection System (Bio-Rad, Marnes-la-Coquette, France). The primer sequences are described in Table 1. PCR reactions were carried out in triplicate at a final volume of 15 µL, containing 7.5 µL MESA Green qPCR Mastermix (Eurogentec, Uppsala, Sweden) and 3.5 µL of cDNA and forward and reverse primers at 300 nM. Thermal cycling conditions were achieved by the activation of DNA polymerase at 95 °C for 10 min, followed by 40 cycles of amplification at 95 °C for 15 s, 60 °C for 30 s, and 72 °C for 30 s. Melting curve analysis was performed to control the absence of non-specific products. For each transcript, the amplification efficiency was determined by the slope of the standard curve generated from twofold serial dilutions of cDNA. The 2^−ΔΔCt^ method was used to determine the relative gene expression. The results are depicted as graphs of relative expression data (fold induction) [41].

### 2.10. Data Analysis

All experimental values are expressed as the average of mean ± standard deviation. The error bars presented on the figures correspond to the standard deviation. Statistical significance was calculated by two-way ANOVA and Tukey’s multiple-comparisons test, with a significance level of *p* ≤ 0.05.

## 3. Results

### 3.1. Effects of Schot and Spina on Cell Viability

**Figure 1:**
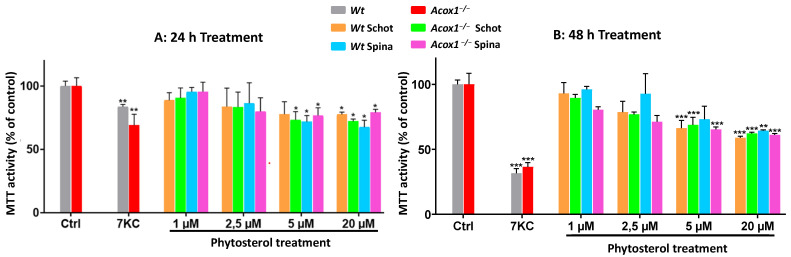
Effects of Schot and Spina on the cell proliferation and viability of the *Wt* and the *Acox1^−/−^* BV-2 microglial cells for 24 (**A**) or 48 (**B**) hours. Cell proliferation and viability were assessed by the MTT test. Cells were treated with Schot or Spina at a range of concentrations from 1 to 20 µM. 7-ketocholesterol (7-KC) was used at 20 µM as a positive control. All compounds were tested for 24 or 48 h. All values are presented as means ± SD of two independent experiments performed in triplicate. Values were normalized to the control. Statistical significance compared to control (*** *p* ≤ 0.001, ** *p* ≤ 0.01, * *p* ≤ 0.05) was determined using one-way ANOVA followed by Tukey’s test.

An MTT test was performed to evaluate the effect of Schot and Spina on the mitochondrial function and cell viability of *Wt* and *Acox1^−/−^* BV-2 microglial cells. The effect of Schot and Spina on the cell viability was assessed at a concentration range of 1 to 20 µM for 24 or 48 h. 7-ketocholesterol (20 µM) was used as a cytotoxic positive control. No cytotoxic effect was shown under either Schot or Spina treatment at 1 or 2.5 µM for 24h (Figure 1A,B). Regarding the genotype, the treatment of both the *Wt* and *Acox1^−/−^* BV-2 cell lines by Schot and Spina showed no significant differences in the effect of the two phytosterols, even at the highest concentration of 20 µM, revealing 75 % viability for treated cells compared to untreated control cells. However, treatment with Schot and Spina for 48 h exhibited a more pronounced effect, with Schot treatment inducing a decrease in cell viability of 40% at 20 µM (Figure 1B).

On the other hand, *Acox1^−/−^* BV-2 cells were shown to be more sensitive to Spina treatment compared to *Wt* BV-2 cells, especially at the 1 and 2.5 µM concentrations. However, the cell viability was still around 80% (Figure 1). Like Schot at 48 h, Spina decreased the viability of *Wt* and *Acox 1^−/−^* BV-2 cells by almost 40% at 20 µM (Figure 1). Moreover, the data obtained after 7-KC treatment revealed that the mitochondrial activity of the *Acox1^−/−^* BV-2 cells was significantly affected by 7-KC treatment at 24 h compared to that of the *Wt* BV-2 cells (Figure 1A). The cell viability of both cell lines was reduced in response to 7-KC treatment at 24 and 48 h (Figure 1A,B). The inhibition of cell viability reached 75% when *Acox 1^−/−^* BV-2 cells were treated with 7-KC for 48 h (Figure 1B). 7-KC is a major toxic and pro-oxidant product of cholesterol oxidation that is known for its cytotoxic effect and its ability to trigger cell death [21].

### 3.2. Schot and Spina Effects on LPS-Induced Intracellular ROS Accumulation

**Figure 2:**
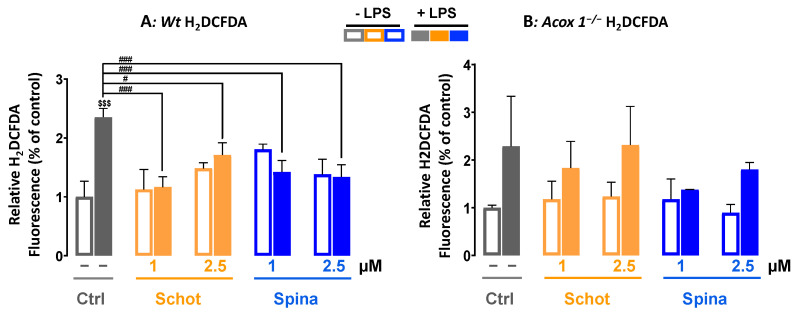
Effects of Schot and Spina on ROS production induced by LPS in the *Wt* (**A**) and *Acox1^−/−^* (**B**) BV-2 microglial cells assessed by the H_2_DCFDA (5 µM) dye test. Cells were incubated for 7 h with Schot or Spina (1 or 2.5 µM) in the absence or presence of LPS (1 µg/mL). All values are presented as means ± SD of two independent experiments performed in triplicate, with the statistical significance of the increase in mean signal indicated as ### *p* ≤ 0.01 and # *p* ≤ 0.05 compared to LPS and $$$ *p* ≤ 0.001 compared to the different treatments with or without LPS administration. Statistical significance was determined using two-way ANOVA followed by Tukey’s test for multiple comparisons.

ROS play a crucial role in cell signaling for growth, differentiation, proliferation, and apoptosis. In this study, we investigated the effect of Schot and Spina at 1 or 2.5 µM on intracellular ROS accumulation induced by LPS treatment at 1 µg/mL. We used two different ROS probes, H_2_DCFDA and DHE. H_2_DCFDA is a specific probe for the detection of H_2_O_2_. H_2_DCFDA can also be oxidized by hydroxyl radicals, hydroperoxides, and peroxynitrite. However, it has been shown to be highly sensitive to H_2_O_2_ [42]. As shown in Figure 2, in contrast to the *Acox1^−/−^* cells, LPS treatment induced significant intracellular ROS production in the *Wt* cells, exhibiting a 2.3-fold increase in LPS-treated cells compared to the control (Figure 2A). This LPS-increased ROS production was significantly abrogated by cotreatment with Schot and Spina at a minimum of 1 µM in *Wt* cells (Figure 2A).

**Figure 3:**
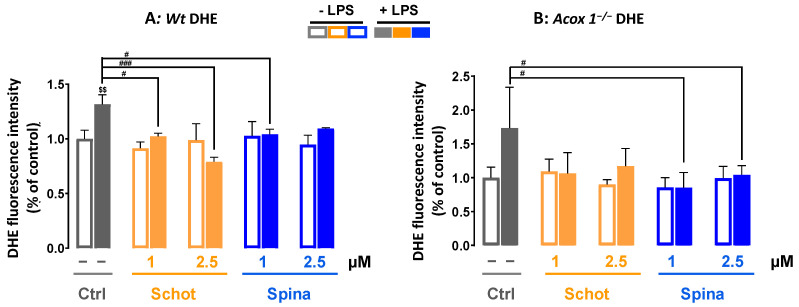
Effect of Schot and Spina on ROS production induced by LPS in the *Wt* (**A**) and *Acox1^−/−^* (**B**) BV-2 microglial cells assessed by the DHE (5 µM) dye test. Cells were incubated for 7 h with Schot or Spina (1 or 2.5 µM) in the presence or absence of LPS (1 µg/mL). All values are presented as means ± SD of two independent experiments performed in triplicate, with the statistical significance of the increase in mean signal indicated as ### *p* ≤ 0.01, # *p* ≤ 0.05 compared to LPS and $$ *p* ≤ 0.01 compared to the different treatments with or without LPS administration. Statistical significance was determined using two-way ANOVA followed by Tukey’s test for multiple comparisons.

The detection of superoxide radicals by DHE probes revealed broadly similar results to the H_2_DCFDA test. The treatment with Schot or Spina alone had no effect on superoxide production. LPS treatment induced significant superoxide radical accumulation in the *Wt* BV- 2 cells (Figure 3A). This effect was significantly attenuated by cotreatment with Schot at 1 or 2.5 µM in a dose-dependent manner and slightly attenuated by Spina at 1 µM compared to LPS-treated cells (Figure 3A). Treatment with Schot or Spina alone had no effect on superoxide production in *Acox1^−/−^* BV-2 cells. Meanwhile, LPS-treated *Acox1^−/−^* BV-2 cells showed no significant increase in superoxide production (Figure 3B).

### 3.3. Schot and Spina Effects on LPS-Induced Nitric Oxide (NO) Generation

**Figure 4:**
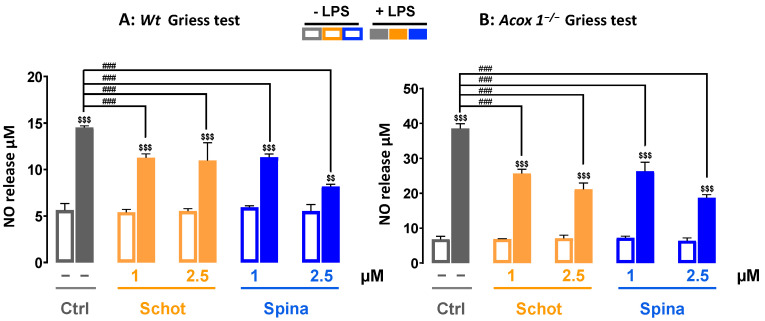
Effect of Schot and Spina on the NO production induced by LPS in the culture media of the *Wt* (**A**) and *Acox1^−/−^* (B) BV-2 microglial cells assessed by the Griess test. Cells were incubated for 24h with Schot or Spina (1 or 2.5 µM) in the absence or the presence of LPS (1 µg/mL). All values are presented as means ± SD of two independent experiments performed in triplicate, with the statistical significance of the increase in mean signal indicated as ### *p* ≤ 0.01 compared to LPS and $$$ *p* ≤ 0.001 and $$ *p* ≤ 0.01 compared to the different treatments with or without LPS administration. Statistical significance was determined using two-way ANOVA followed by Tukey’s test for multiple comparisons.

Nitric oxide is a signaling molecule known for its important role in acute and chronic inflammation in the nervous system and apoptosis [43]. To evaluate the anti-inflammatory effects of Schot and Spina, we measured the level of NO production in the absence or the presence of LPS. Treatment with Schot or Spina alone at 1 or 2.5 µM had no effect on NO production in *Wt* BV-2 cells (Figure 4A). After incubation with LPS, the level of NO was increased significantly by three folds in comparison to the control cells (Figure 4A). Both Schot and Spina were able to significantly reduce the LPS-induced NO level (Figure 4A). Similar effects were observed in the *Acox 1^−/−^* BV-2 cell line on the LPS-induced NO level after treatment with Schot or Spina (Figure 4B).

### 3.4. Effect of Schot and Spina on Peroxisomal Catalase and ACOX1 Activities

**Figure 5:**
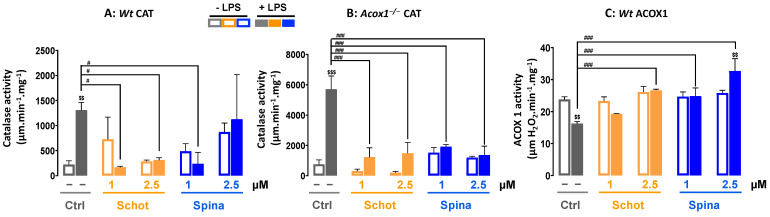
Effect of Schot and Spina on catalase activity in the *Wt* (**A**) and *Acox1^−/−^* (**B**) BV-2 microglial cells, and on ACOX1 activity (**C**) in *Wt* BV-2 microglial cells. Cells were incubated for 24 h with Schot or Spina (1 or 2.5 µM) in the absence or the presence of LPS (1 µg/mL). All values are presented as means ± SD of two independent experiments performed in triplicate, with the statistical significance of the increased mean signal indicated as ### *p* ≤ 0.01 and # *p* ≤ 0.05 compared to LPS and $$$ *p* ≤ 0.001 and $$ *p* ≤ 0.01 compared to the different treatments with or without LPS administration. Statistical significance was determined using two-way ANOVA followed by Tukey’s test for multiple comparisons.

We investigated the effect of Schot and Spina on peroxisomal LPS-dysregulated function by measuring the activities of peroxisomal catalase and ACOX1. As shown in Figure 5, treatment with either Schot or Spina at both concentrations had no significant effect on catalase or ACOX1 activities. On the other hand, LPS treatment significantly increased (>6 folds) the CAT activity in the *Wt* BV-2 cells and even more so (>7.5 folds) in the *Acox1^−/−^* BV-2 cells (Figure 5A,B). Previously, we reported that the deficiency of ACOX1 in BV-2 microglial cells is accompanied by increased catalase activity [19]. Interestingly, in both *Wt* and *Acox1^−/−^* cells, both Schot (1 and 2.5 µM) and Spina (at least 1 µM) treatments were able to restore the LPS-induced CAT activity to the control level (Figure 5A,B). Peroxisomal ACOX1 activity was significantly reduced by LPS treatment in the *Wt* BV-2 cells, and both Schot and Spina were able to restore the ACOX1 activity in *Wt* cells (Figure 5C). Thus, argan-oil-derived phytosterols can counteract the deleterious effects of LPS on peroxisomal antioxidant and β-oxidative activities.

### 3.5. Effects of Schot and Spina on Peroxisomal Protein Expression

**Figure 6:**
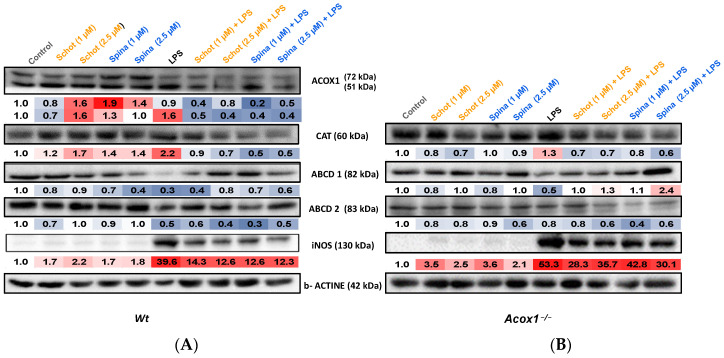
Immunoblotting assay showing effects of Schot and Spina on the expression of the peroxisomal proteins ACOX1, CAT, ABCD1, and ABCD2 and the inflammatory marker iNOS in *Wt* (**A**) and *Acox1^−/−^* (**B**) BV-2 microglial cells. Cells were incubated for 24 h with Schot or Spina (1 or 2.5 µM) in the absence or presence of LPS (1 µg/mL). Cell homogenates were analyzed by PAGE-SDS electrophoresis and subjected to immunoblotting. Band intensities were analyzed by densitometry and standardized to β-actin expression level. Table values represent standardized densitometric analysis obtained after the signal intensity quantification of different proteins.

To investigate the role of Schot and Spina treatment in LPS-induced peroxisomal function dysregulation and inflammation, we evaluated the expression of the proteins involved in peroxisomal antioxidative (catalase) and β-oxidative (ACOX1, ABCD1, and ABCD2) functions and acting as inflammation markers (iNOS) in *Wt* and *Acox1^−/−^* BV-2 microglial cells. Treatment with Schot and Spina both increased ACOX1 polypeptide levels in *Wt* cells (Figure 6A), while LPS increased only the expression of the ACOX1 51 kDa peptide, which resulted from 72 kDa polypeptide processing [40]. The combined treatments of Schot–LPS and Spina–LPS both substantially decreased ACOX1 peptide expression in *Wt* BV- 2 cells (Figure 6A). On the other hand, incubation with LPS increased the catalase (by 2.2 and 1.3 folds) and iNOS (39.6 and 53.3 folds) protein expression in *Wt* and *Acox1^−/−^* BV-2 cells, respectively, compared to control cells (Figure 6A,B). These increases seemed to be attenuated by Schot or Spina cotreatment, with Schot at 1 µM bringing the catalase protein expression level close to the control value in the *Wt* BV-2 cell line. Both Schot and Spina exhibited high efficiency in decreasing LPS-induced iNOS expression in *Wt* BV-2 cells, but only very slightly in *Acox1^−/−^* BV-2 cells (Figure 6A,B). The treatment with Schot or Spina alone had a slight increasing effect on catalase protein expression in Wt BV-2 cells, while in *Acox1^−/−^* BV-2 cells, both phytosterols showed only weak effects (Figure 6A,B).

Further, LPS treatment decreased the expression of both peroxisomal VLCFA transporters, ABCD1 and ABCD2, in *Wt* and *Acox1^−/−^* BV-2 cells (Figure 6A,B). Interestingly, the combined treatment of LPS with Schot or Spina abrogated, fully in *Acox1^−/−^* cells and partially in *Wt* cells, the effect of LPS on ABCD1 protein expression. While at the higher concentration of 2.5 µM, both phytosterols increased ABCD1 expression level in the LPS-treated *Acox1^−/−^* cell line compared to the control cells (Figure 6B). However, neither Schot nor Spina rescued the LPS-induced decrease in the ABCD2 protein expression level in either cell line (Figure 6A,B).

### 3.6. Shot and Spina Effect on the Expression of Peroxisomal Protein-Encoding Genes

**Figure 7:**
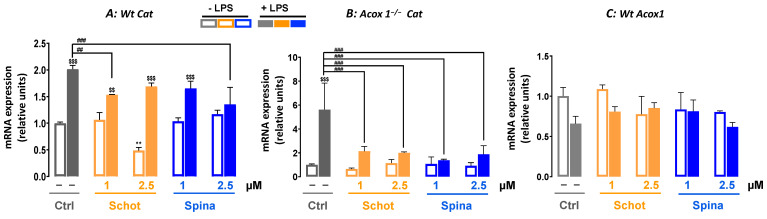
Effect of Schot and Spina treatment on the gene expression of *Cat* (**A**,**B**) and *Acox1* (**C**) in *Wt* (**A**,**C**) and *Acox1^−/−^* (**B**) BV-2 microglial cells. Cells were incubated for 24 h with Schot or Spina (1 or 2.5 µM) in the absence or presence of LPS (1 µg/mL). Total RNA was isolated from BV-2 cells, and then the expression level of genes of interest was quantified by real-time RT-qPCR. All values are presented as means ± SD of two independent experiments performed in triplicate, with the statistical significance of the increased mean indicated as ** *p* ≤ 0.01 compared to control; ### *p* ≤ 0.001 and ## *p* ≤ 0.01 compared to LPS and $$$ *p* ≤ 0.001 and $$ *p* ≤ 0.01 compared to the different treatments with or without LPS administration. Statistical significance was determined using two-way ANOVA followed by Tukey’s test for multiple comparisons.

**Figure 8:**
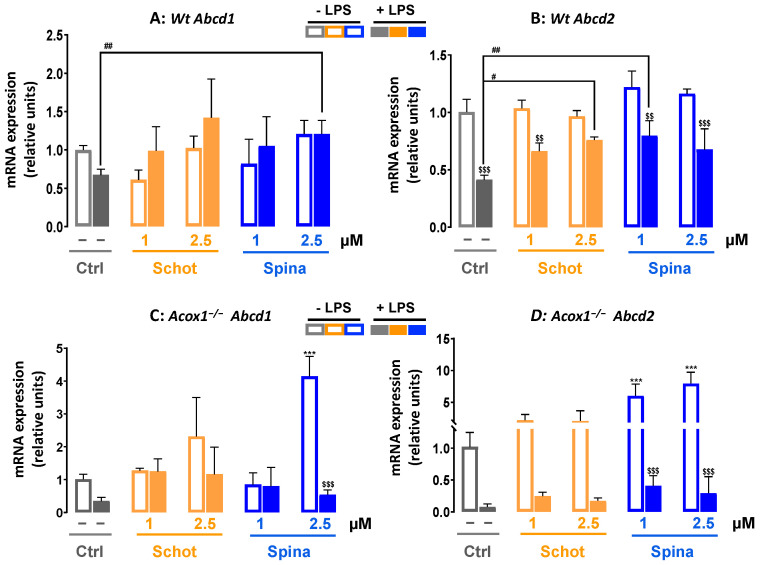
Effect of Schot or Spina treatment on the gene expression of *Abcd1* (**A**,**C**) and *Abcd2* (**B**,**D**) in *Wt* (**A**,**B**) and *Acox1^−/−^* (**C**,**D**) BV-2 microglial cells. Cells were incubated for 24h with Schot or Spina (1 or 2.5 µM) in the absence or presence of LPS (1 µg/mL). Total RNA was isolated from BV-2 cells, and then the expression level of genes of interest was quantified by real-time RT-qPCR. All values are presented as means ± SD of two independent experiments performed in triplicate, with the statistical significance of the increased mean signal indicated as *** *p* ≤ 0.001 compared to control; ## *p* ≤ 0.01 and # *p* ≤ 0.05 compared to LPS; and $$$ *p* ≤ 0.001 and $$ *p* ≤ 0.01 compared to the different treatments with or without LPS administration. Statistical significance was determined using two-way ANOVA followed by Tukey’s test for multiple comparisons.

LPS treatment significantly increased the *Cat* mRNA level in both *Wt* and *Acox1^−/−^* cells (Figure 7A,B). Only the cotreatments of Schot–LPS at 1 µM and Spina–LPS at 2.5 µM caused a significant decrease in LPS-induced *Cat* gene expression in *Wt* cells (Figure 7A). In *Acox1^−/−^* cells, the LPS-induced increase in *Cat* mRNA expression was mostly abrogated by Scot and Spina cotreatment (Figure 7B). The gene expression of *Acox1* was assessed in the *Wt* cell line, showing no significant changes after treatment with either Scot or Spina alone or in combination with LPS (Figure 7A). On the other hand, only the combination of LPS and Spina at 2.5 µM increased *Abcd1* expression (Figure 8A). LPS treatment significantly reduced the gene expression of *Abcd2* mRNA in *Wt* BV-2 cells (Figure 8B). Although neither Schot nor Spina alone had an effect on *Abcd1* and *Abcd2* mRNA levels in *Wt* cells, in the presence of LPS, only the *Abcd2* gene expression level was increased by both phytosterols (Figure 8B). In *Acox1^−/−^* BV-2 cells, the reduction in *Abcd1* and *Abcd2* mRNA by LPS was not significant (Figure 8C,D). Schot alone or in the presence of LPS also had no effect on *Abcd1* or *Abcd2* mRNA levels (Figure 8C,D). In contrast, Spina alone increased the gene expression of both *Abcd1* (at 2.5 µM) and *Abcd2* (at 1 µM and 2.5 µM), and in the presence of LPS, Spina slightly increased the mRNA expressions of both genes in *Acox1^−/−^* cells (Figure 8C,D).

### 3.7. Effect of Schot and Spina on the Expression of Inflammation-Marker-Encoding Genes

**Figure 9:**
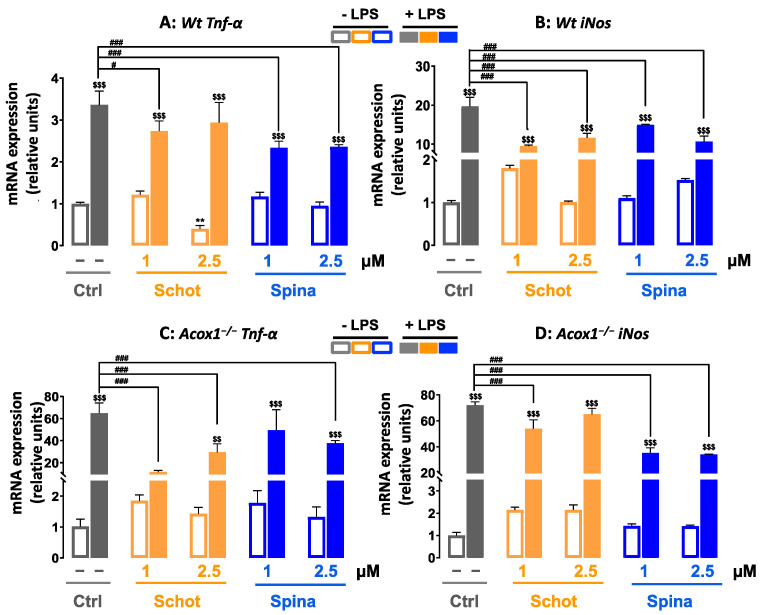
Effect of Schot and Spina treatments on the gene expression of the proinflammatory markers *Tnf-α* (**A**,**C**) and *iNos* (**B**,**D**) in *Wt* (**A**,**B**) and *Acox1^−/−^* (**C**,**D**) BV-2 microglial cells. Cells were incubated for 24 h with Schot or Spina (1 or 2.5 µM) in the absence or presence of LPS (1 µg/mL). Total RNA was isolated from BV-2 cells, and then the expression level of genes of interest was quantified by real-time RT-qPCR. All values are presented as means ± SD of two independent experiments performed in triplicate, with the statistical significance of the increased mean signal indicated as ** *p* ≤ 0.01 compared to control; ### *p* ≤ 0.01 and # *p* ≤ 0.05 compared to LPS; $$ *p* ≤ 0.01 and $$$ *p* ≤ 0.001 compared to the different treatments with or without LPS administration. Statistical significance was determined using two-way ANOVA followed by Tukey’s test for multiple comparisons.

In response to LPS treatment, the mRNA expression levels of *Tnf-α* and *iNos* were increased by more than 3 and 20 folds, respectively, in the microglial *Wt* cells (Figure 9A,B). The combined treatments of Schot–LPS and Spina–LPS at both concentrations significantly attenuated the LPS-induced *Tnf-α* and *iNos* mRNA level increases (Figure 9A,B). In the *Acox1^−/−^* cell line, the cotreatments of Schot–LPS and Spina–LPS limited the increased mRNA levels of *Tnf-α* and *iNos* (Figure 9C,D).

**Figure 10:**
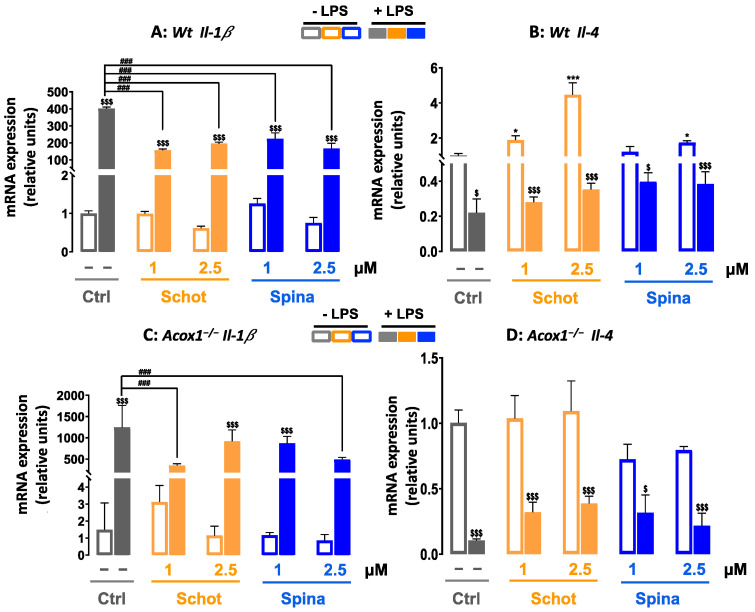
Effect of Schot and Spina treatment on the gene expression of the proinflammatory marker *Il-b* (**A**,**B**) and the anti-inflammatory marker *Il-4* (**C**,**D**) in the *Wt* (**A**,**C**) and *Acox1^−/−^* (**B**,**D**) BV-2 microglial cells. Cells were incubated for 24 h with Schot or Spina (1 or 2.5 µM) in the absence or presence of LPS (1 µg/mL). Total RNA was isolated from BV- 2 cells, and then the expression level of genes of interest was quantified by real-time RT-qPCR. All values are presented as means ± SD of two independent experiments performed in triplicate, with the statistical significance of the increased mean signal indicated as *** *p* ≤ 0.001 and * *p* ≤ 0.05 compared to control; ### *p* ≤ 0.01 and compared to LPS; and $$$ *p* ≤ 0.001 and $ *p* ≤ 0.05 compared to the different treatments with or without LPS administration. Statistical significance was determined using two-way ANOVA followed by Tukey’s test for multiple comparisons.

In addition to the enhancement of *Tnf-α* and *iNos* gene expression, LPS also strongly increased the mRNA level of the proinflammatory cytokine *Il-1β* in both the Wt and *Acox1^−/−^* cell lines. These LPS-dependent increases were significantly attenuated by cotreatment with Schot or Spina at both concentrations (Figure 10A,B). In contrast, treatment with Schot or Spina alone, at both concentrations, had no effect on *Il-1β* expression in the *Wt* and *Acox1^−/−^* cell lines when compared to the control cells (Figure 10A,B). On the other hand, we also evaluated the gene expression of the anti-inflammatory cytokine *Il-4*. The obtained data revealed that Schot and Spina treatment increased the *Il-4* mRNA expression in the *Wt* cells but not in the *Acox1^−/−^* cells (Figure 10C,D). However, the LPS-reduced levels of *Il-4* mRNA in both cell lines were partially but significantly rescued by Schot and Spina treatment at concentrations of 1 and 2.5 µM (Figure 10C,D).

## 4. Discussion

Microglial cells are considered resident macrophages in the central nervous system, and they play a key role in brain infections and inflammation [44]. The chronic activation of microglial cells induces neuronal damage and loss, which lead to the development of several neurodegenerative diseases [45]. The control of microglia activation has a pivotal role in blocking the development of neurodegenerative disorders and attenuating their progression [46]. In recent decades, plant-derived compounds with pharmacological activities have received considerable attention from researchers as a potential source of new alternative drugs for treating neurological disorders.

In this study, we evaluated the effect of two phytosterols, Schot and Spina, on LPS-induced inflammation in Wt and Acox1^−/−^ murine microglia BV-2 cell lines. Here, we showed that at low concentrations, Schot and Spina had no effect on Wt and Acox1^−/−^ BV- 2 viability. Accordingly, in a previous study, we showed that Schot and Spina from argan oil were not toxic to microglial BV-2 cells and could impact the mitochondrial membrane potential [22]. However, we noted and confirmed that the viability of both cell lines was significantly altered by 7-KC treatment, which may lead to cell death by oxiapoptophagy, including oxidative stress and the induction of death by apoptosis associated with autophagy [47].

Furthermore, we evaluated the total ROS production by H_2_DCFDA, which is largely used as a hydrogen peroxide (H_2_O_2_)-specific probe but can detect a wide range of ROS, such as superoxide anions (O2^•−^), hydroxyl radicals (^•^OH), and peroxynitrite (ONOO^−^) [48]. We also used the DHE probe to detect superoxide radicals. Mitochondrial respiration represents the main source of superoxides, which are the principal ROS in the central nervous system [49]. Our results revealed the strong enhancement of ROS production by Wt BV-2 microglial cells in response to LPS treatment. The activation of microglia by LPS, which triggers high ROS production [50], may initiate apoptosis and disrupt the blood–brain barrier (BBB), leading to brain function damage that may be irreversible [51]. Oxidative stress has been widely implicated in the development of several neurodegenerative disorders [52,53,54]. The increased ROS generation was significantly inhibited by both phytosterols (Schot and Spina). In their study, Yoshida and Niki showed that phytosterols chemically act as antioxidants and radical scavengers [55]. Moreover, both Schot and Spina attenuated the activity and mRNA and protein expression of catalase induced by LPS. Catalase constitutes the main peroxisomal antioxidant H_2_O_2_-degrading enzyme, and the perturbation of catalase activity is related to many neurodegenerative diseases [56]. The regulation of catalase expression is controlled at both the mRNA and protein levels. The control of catalase activity can also be related to its post-translational modifications. CAT activity is increased by phosphorylation at Ser167 by protein kinase C delta [57] or at both Tyr231 and Tyr386 by the Abelson tyrosine-protein kinases ABL1 and ABL2 [58]. Further, we have previously shown that argan oil regulates the mRNA level and activity of catalase induced by LPS in the mice brain [20]. The high content of Schot and Spina in argan oil suggests that they may act as antioxidants, preventing H_2_O_2_ microglial accumulation and modulating the activity of antioxidant enzymes, which also act as ROS scavengers [59,60].

Furthermore, we revealed that LPS treatment increased, in both Wt and Acox1^−/−^ BV-2 cells, NO release in the culture medium concomitantly with the increased mRNA and protein expression of iNOS. Under normal conditions, iNOS is weakly expressed in the brain. However, after LPS treatment, microglial cells constitute the main source of iNOS and NO [61,62]. NO, produced by iNOS, plays a key signaling role in neurotransmission and neuroinflammation and mediates neuron and glial cell interactions in the brain [62]. The NO released from activated microglia inhibits the reuptake of glutamate at the presynaptic site, blocking the activation of N-Methyl-D-aspartate (NMDA) receptors and leading to neuronal death [63]. Our results were in accordance with the previous reports suggesting that phytosterols decrease NO production [64].

Lipid metabolism, including peroxisomal metabolism, is crucial for neuronal development, synaptic plasticity, and microglial function [65,66]. The perturbation of lipid metabolism at the level of synthesis, transport, or catabolism contributes to the pathogenesis of several neurodegenerative disorders, including Alzheimer’s disease [67], Parkinson’s disease [68], and peroxisomal leukodystrophies [69]. ACOX1 deficiency is a severe peroxisomal neurological disorder with early developmental regression, a loss of vision and hearing, and death between 4 and 10 years of age [70]. ACOX1 deficiency causes an accumulation of VLCFA and glial loss within the brain [70,71]. ACOX1 is known as the rate-limiting enzyme of peroxisomal fatty acid β-oxidation [72]. Here, we showed that the LPS treatment of microglial *Wt* cells downregulated ACOX1 expression at the enzymatic level but not at the mRNA level. Such results are in accordance with previously reported data in mice livers [73]. Such a discrepancy between translational and transcriptional regulation can be explained by differences in mRNA and protein decay rates, while abnormal translation can accelerate mRNA decay [74]. The control of protein translation is governed by the PERK/eIF2α-P/ATF4 signaling axis, which abrogates the decline in protein synthesis during endoplasmic reticulum stress initiated by LPS [75]. Furthermore, LPS has been reported to reduce Sirtuin 3 (SIRT3) expression and activity through TLR4 [76]. Interestingly, the knockdown of deacetylase SIRT3 led to the downregulation of ACOX1 activity. Such SIRT3-dependent ACOX1 regulation was shown to involve an interaction between Heat shock protein 70 and ACOX1 [77].

VLCFA-CoA esters are imported into the peroxisome thanks to two ABCD transporters (i.e., ABCD1 and ABCD2). Notably, ABCD1 deficiency is the principal cause of the inherited peroxisomal disorder X-linked adrenoleukodystrophy (X-ALD), associated with neurodegeneration and inflammatory cerebral demyelination [17]. ABCD2 is a sterol-regulatory-element-binding protein target gene, and its gene expression is induced by sterols [78]. Here, we revealed that both phytosterols, Schot and Spina, induced *Abcd2* gene expression and were able to counteract the negative effect of LPS. The ABCD2 gene is the closest homolog of ABCD1 [69], and its upregulation compensates for ABCD1 deficiency in X-ALD skin fibroblasts [79,80]. This functional redundancy has been investigated as a new alternative pharmacotherapy for X-ALD [81,82]. Interestingly, Nomaguchi et al. showed that *Aloe vera* phytosterols activated mouse hepatic PPARα and its target genes, including *Acox1*, in a dose-dependent manner [83]. The activation of PPARα is dependent on several factors (fatty acids, hormone release, cytokines, and growth factors) [84]. In addition, certain natural ligands, such as the polyphenolic compound resveratrol, ferulic acid, and the oxidized derivative of campesterol, are active biological modulators of several signaling proteins, including PPARα [16,85,86]. PPARα regulates target genes that are involved in glucose and peroxisomal fatty acid oxidation, as well as signaling pathways modulating inflammation [61]. Recently, Spinasterol was demonstrated to increase the protein expression of PPARγ [87] and stigmasterol-attenuated inflammation through the butyrate-PPARγ axis [88]. PPARs exert their anti-inflammatory effect through inhibiting the gene expression of the proinflammatory transcription factors, including the signal transducer and activator of transcription activator protein-1 and nuclear factor NF-κB [62].

LPS-activated microglia cells also express other inflammatory mediators, such as IL- 1β and TNF-α, which promote proinflammatory cytokine generation [89]. We revealed that Schot and Spina significantly attenuated, in both *Wt* and *Acox1^−/−^* BV-2 microglial cell lines, the LPS-induced mRNA expression of *Tnf-α* and *Il-1β*. Several studies have reported that LPS-stimulated microglia induced the production of inflammatory mediators, such as NO, TNF-α, and IL-1β [90]. However, during acute inflammation, TNF-α plays a critical role in restoring brain homeostasis, fighting against brain injury and neurodegeneration [63].

In our experiments, cotreatment with Schot or Spina attenuated the LPS-induced expression of the inflammatory mediators *Tnf-α* and *Il-1β*. These results indicated that Schot and Spina had anti-inflammatory effects on LPS-stimulated BV2 microglial cells. Interestingly, the LXR nuclear receptors, considered as integrators of metabolic and inflammatory signaling [64], can be modulated by phytosterols, including Schot and Spina [22]. Additionally, sitosterol, as with several other phytosterols, triggers an anti-inflammatory response by downregulating several components (i.e., NO, *iNos*, and *Tnf-α*) of the TLR4 pathway [91]. In a clinical trial, Kurano et al. [92] highlighted the negative correlation between sitosterol levels and circulating TNF-α and IL-6 levels. In our study, we also found that Schot and Spina partially but significantly re-established the LPS-reduced expression *Il-4* mRNA. As a pleiotropic anti-inflammatory cytokine, IL-4 controls brain homeostasis, including tissue repair and cellular protection, upon microglia activation in neuroinflammation [93]. Il-4 suppresses the liberation of the pro-inflammatory factors, such as IL-1β, IL-6, TNF-α, and NO [94,95]. In addition, in vitro and in vivo, IL-4 can cause activated microglia to move towards a regenerative and anti-inflammatory phenotype [96,97].

## 5. Conclusions

Collectively, our data highlighted the protective effects of the AO phytosterols Schottenol and Spinasterol against LPS-induced microglia oxidative stress, inflammation, and peroxisome dysfunction through lowering oxidative and nitrosative stress and pro-inflammatory gene expression and normalizing the peroxisomal antioxidant catalase expression and fatty acid β-oxidation functions. Our results may provide new evidence supporting the health-promoting properties of argan oil’s bioactive molecules, including the antioxidant and anti-inflammatory activities of phytosterols.

## Figures and Tables

**Table 1:** Sequences of the primers used for qPCR.

Gene Name	Accession Number	Primer Sequences
*Abcd1-F* *Abcd1-R*	NM_007435.2	5′GCCAAGTTGTGGATGTGGAG3′ 5′TTCCGCAGAGTCGGGATAGA3′
*Abcd2-F* *Abcd2-R*	NM_011994.4	5′TAGAACGCATCCTGCACAGC3′ 5′CTCCTTCGCCATCGAATTGT3′
*Acox1-F* *Acox1-R*	NM_001377522.1	5′TCGAAGCCAGCGTTACGAG3′ 5′GGTCTGCGATGCCAAATTCC3′
*Cat-F* *Cat-R*	NM_009804.2	5′AGCGACCAGATGAAGCAGTG3′ 5′TCCGCTCTCTGTCAAAGTGTG3′
*Il-1β-F* *Il-1β-R*	NM_008361.4	5′GAGATTGAGCTGTCTGCTCA 3′ 5′AAGGAGAACCAAGCAACGAC 3′
*Il-4-F* *IL-4-R*	NM_021283.2	5′CCATATCCACGGATGCGACAA3′ 5′CCTCGTTCAAAATGCCGATGAT3′
*iNos-F* *iNos-R*	NM_010927.4	5′CCTAGTCAACTGCAAGAGAA3′ 5′TTTCAGGTCACTTTGGTAGG3′
*Tnf-α-F* *Tnf-α-R*	NM_013693.3	5′CCCTCACACTCAGATCATCTTCT3′ 5′GCTACGACGTGGGCTACAG3′
*36b4-F* *36b4-R*	NM_007475.5	5′CGACCTGGAAGTCCAACTAC3′ 5′ATCTGCTGCATCTGCTTG3′

## Data Availability

Data are contained within the article.
